# Maternal inflammatory, lipid and metabolic markers and associations with birth and breastfeeding outcomes

**DOI:** 10.3389/fnut.2023.1223753

**Published:** 2023-09-04

**Authors:** Sophie Hilario Christensen, Ane Lilleøre Rom, Tine Greve, Jack Ivor Lewis, Hanne Frøkiær, Lindsay H. Allen, Christian Mølgaard, Kristina Martha Renault, Kim F. Michaelsen

**Affiliations:** ^1^Department of Nutrition, Exercise and Sports, Faculty of Science, University of Copenhagen, Copenhagen, Denmark; ^2^Department of Obstetrics, Copenhagen University Hospital, Rigshospitalet, Denmark; ^3^Research Unit of Gynaecology and Obstetrics, Department of Clinical Research, University of Southern Denmark, Odense, Denmark; ^4^Department of Obstetrics and Gynecology, Copenhagen University Hospital, Hvidovre Hospital, Hvidovre, Denmark; ^5^Department of Veterinary and Animal Science, Faculty of Health and Medical Sciences, University of Copenhagen, Copenhagen, Denmark; ^6^USDA, ARS Western Human Nutrition Research Center, Davis, CA, United States; ^7^Department of Clinical Medicine, Faculty of Health and Medical Sciences, University of Copenhagen, Copenhagen, Denmark

**Keywords:** inflammatory markers, lipid markers, metabolic markers, *in utero* programming, pregnancy, breastfeeding, human milk composition

## Abstract

**Background:**

Conditions *in utero* influence intrauterine and postnatal infant growth and a few studies indicate that maternal inflammation and insulin resistance might affect birth and breastfeeding outcomes. Furthermore, hormones in human milk (HM) may influence infant appetite-regulation and thereby milk intake, but the associations are less understood.

**Objective:**

(1) To investigate associations between maternal inflammatory, lipid and metabolic markers and birth and breastfeeding outcomes, and (2) to assess predictors of maternal inflammatory, lipid and metabolic markers in pregnancy.

**Methods:**

Seventy-one mother-infant dyads participating in the Mothers, Infants and Lactation Quality (MILQ) study were included in the present study. Fasting blood samples were collected around 28th gestational week, and HM samples at three time points from 1.0 to 8.5 months, where milk intake was assessed using 24-h test weighing. Maternal plasma inflammatory, lipid and metabolic markers included high-sensitive C-reactive protein (hs-CRP), tumor-necrosis factor-α (TNFα), interferon-γ (IFNγ), Interleukin (IL)-6, IL-8, high-, low-, and very-low-density lipoprotein (HDL, LDL, VLDL), total-cholesterol, triglycerides, leptin, adiponectin, insulin, C-peptide, the homeostasis model assessment of insulin resistance (HOMA-IR) and glucose concentration at *t* = 120 min following an oral glucose tolerance test. Of these, TNFα, IFNγ, IL-6, IL-8, leptin, adiponectin and insulin were also measured in HM samples.

**Results:**

HDL in pregnancy was inversely associated with gestational age (GA) at birth and GA-adjusted birthweight z-score, whereas triglycerides and glucose (*t* = 120) were positively associated with GA-adjusted birthweight z-score. Higher hs-CRP, VLDL and triglycerides were associated with a higher placental weight. Furthermore, higher HDL, insulin, leptin and HOMA-IR were associated with longer duration of exclusive breastfeeding (EBF). Higher pre-pregnancy BMI was the main predictor of higher levels of hs-CRP, log-TNFα, leptin, insulin, C-peptide, and HOMA-IR.

**Conclusion:**

Maternal lipid and metabolic markers influenced birthweight z-score and placental weight as well as duration of EBF. Furthermore, pre-pregnancy BMI and maternal age predicted levels of several inflammatory and metabolic markers during pregnancy. Our findings indicate that maternal lipid and metabolic profiles in pregnancy may influence fetal growth and breastfeeding, possibly explained by overweight and/or higher placental weight.

**Clinical trial registration:**

https://clinicaltrials.gov/, identifier NCT03254329.

## 1. Introduction

Since the 1990s, Barkers theory of *in utero* programming has been well-established proposing that malnutrition during critical windows in fetal life predispose the offspring to increased risk of later disease (1, 2). Primarily low birthweight and small abdominal circumference at birth tend to be associated with higher infant total-cholesterol and lipoprotein levels in adulthood (3). These results are independent of gestational age (GA) at birth indicating that restricted fetal growth rather than premature birth affects plasma lipid levels. Through programming mechanisms causing appetite-regulation and energy homeostasis to become dysregulated during restricted intrauterine growth and following catch-up growth (4, 5), the infant is predisposed to an increased risk of later disease such as obesity and type 2 diabetes (6–9). This increased risk may persist throughout the life course and perhaps onto the next generation.

During pregnancy, the fetus has direct access to nutrients from the maternal circulation through the placenta. As such, maternal circulation represents the complete nutrient source for the fetus and concurrently reflects maternal nutritional and health status. Furthermore, obesity during pregnancy is associated with increased levels of C-reactive protein (CRP), interleukin (IL)-6, tumor-necrosis factor-α (TNFα) and leptin (10, 11). Higher levels of cytokines and CRP in women with normal weight or slight overweight have independently been associated with low birthweight (12, 13). Additionally, Swanson and colleagues found among an American population, that the increase in pre-pregnancy body mass index (BMI) from 1995 to 2004 was positively correlated with the increased placental weight in the same period (14). While a healthy pregnancy involves a slight increase in cytokine levels altering insulin sensitivity for the benefit of the growing fetus (15, 16), an excessive increase in these hormones might influence birth outcomes with unintended consequences possibly mediated through effects on the placenta.

Lower breastfeeding rates are seen for overweight and obese mothers and one of the possible explanations includes altered inflammatory and hormonal profiles which may interrupt breastfeeding (17, 18). Gestational diabetes mellitus (GDM) has additionally been proposed as an important risk factor for delayed or unsuccessful breastfeeding (19, 20) and even mild gestational hyperglycemia as a result of a healthy pregnancy may predict a shortened duration of breastfeeding (21). In Denmark, in the period of inclusion for the present study, GDM was diagnosed by a two-hour glucose level of ≥9.0 mmol/L following a 75 g oral glucose dose, i.e., an oral glucose tolerance test (OGTT) (22). Additionally, a homeostasis model assessment of insulin resistances (HOMA-IR) can identify pregnant women at risk of developing GDM, with higher HOMA-IR increasing the risk of GDM (23). Ley et al. reported positive associations between fasting glucose and HOMA-IR measured in pregnancy and HM insulin at 95 days postpartum indicating a larger window for a potential effect on the infant (24). Recent studies further indicate that the early cessation of EBF seen for mothers with overweight and/or GDM might be explained by altered glucose homeostasis and subsequent insulin resistance in pregnancy (18, 25, 26). Additionally, Walker et al. recently found increased hs-CRP and TNFα concentrations in HM of mothers with very low compared to normal milk output and suggested that TNFα inhibits fatty acid uptake in the mammary gland resulting in reduced milk production (27). As such, elevated levels of inflammatory, lipid and metabolic markers in pregnancy due to overweight may exert influence via the placenta as well as through the breastfeeding period, however, the evidence within a healthy population is sparse.

We aimed (1) to investigate associations between maternal inflammatory, lipid and metabolic markers and pregnancy and breastfeeding outcomes within a healthy population, and (2) to assess predictors of inflammatory, lipid and metabolic markers in pregnancy. We hypothesize that concentrations of inflammatory, lipid and metabolic markers are associated with birth outcomes such as placenta weight and birth weight z-score and breastfeeding outcomes such as duration of EBF and HM intake.

## 2. Methods and materials

### 2.1. Study design and participants

We included a subgroup of participants from the Mothers, Infants and Lactation Quality (MILQ) Study (28). The MILQ study is a multi-center cohort study including 1,000 mother-infant dyads and with the aim of developing reference values for micro-and macronutrient concentrations in HM. Data are collected in four sites (Bangladesh, Brazil, Denmark and The Gambia) of which data from Denmark are used in the present analysis.

Pregnant women less than 28 weeks of gestation were invited to participate, and informed consent was obtained. The study was conducted from February 2018 to December 2019 and took place at the Copenhagen University Hospitals, Rigshospitalet and Hvidovre Hospital, as well as the Department of Nutrition, Exercise and Sports, University of Copenhagen.

Women were screened according to the following inclusion criteria for the MILQ study; being non-smokers and 18–40 years old with a pre-pregnancy BMI between 18.5 and 29.9 kg/m^2^. They should have a low intake of fortified foods and only take vitamin-and mineral supplements recommended by the Danish Health Authorities. They were excluded if they expected twins or had preeclampsia, GDM and/or anemia. The latter was accepted if they were willing to take iron supplements.

### 2.2. Data collection

**Figure 1:**
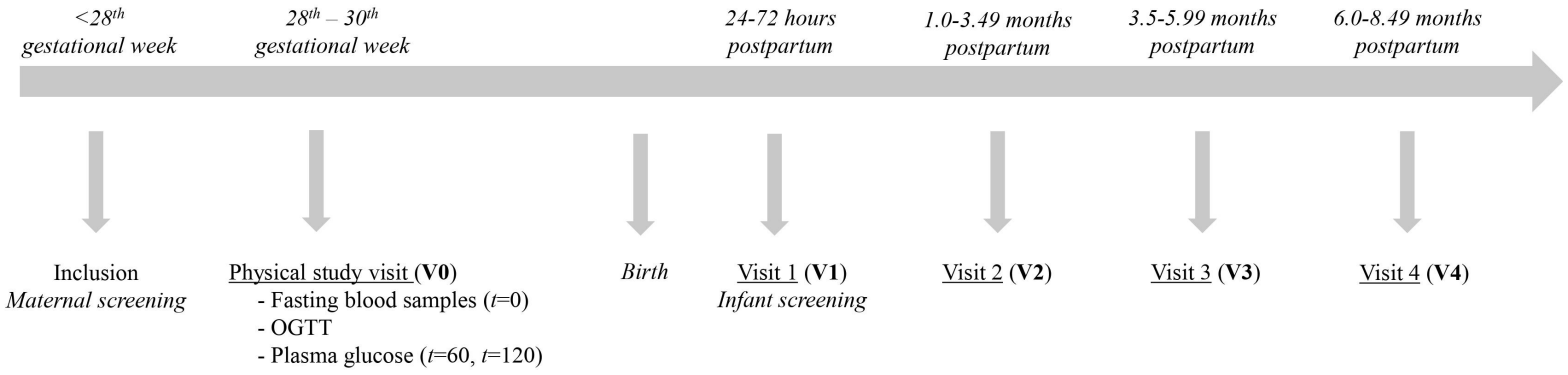
Study design and timeline of participation with collection at four study visits. GA = Gestational Age; OGTT = Oral Glucose Tolerance Test.

Participants attended one physical examination visit during gestational weeks 28–30 (Visit 0, V0), which included fasting blood samples at *t* = 0 and plasma glucose at *t* = 60 and *t* = 120 min following a 75 g oral glucose load (OGTT). Screening of the infants according to MILQ criteria took place 2–3 weeks after birth (Visit 1, V1), and mother-infant dyads followed the protocol of the MILQ study if eligible. The three postpartum examination visits of the MILQ study took place during the periods 1–3.49 months (Visit 2, V2), 3.5–5.99 months (Visit 3, V3) and 6–8.49 months (Visit 4, V4) postpartum (Figure 1). Furthermore, mother-infant dyads were excluded from the MILQ study if they were not exclusively breastfeeding (EBF) at V2, or had ceased breastfeeding at V3.

### 2.3. Sample collection and analyses

Fasting blood samples were collected at V0 and stored at −80°C until analysis. The pregnancy markers included hs-CRP, TNFα, IFNγ, IL-6, IL-8, LDL, HDL, VLDL, total cholesterol, triglycerides, leptin, adiponectin, insulin and C-peptide and were analyzed at Department of Clinical Biochemistry, Copenhagen University Hospital, Hvidovre, Denmark. The markers hs-CRP, LDL, VLDL, HDL, total-cholesterol, triglycerides, insulin and C-peptide were analyzed using Elecsy Reagents (Roche Cobas^®^, F. Hoffmann-La Roche Ltd., Rotkreuz, Switzerland), whereas TNFα were analyzed using DRG^®^ TNF-α ELISA Kit (DRG International Inc., United States), IFNγ and IL-8 using the Invitrogen™ Human IFN gamma and IL-8 Ultrasensitive ELISA Kit (Thermo Fisher Scientific Inc., MA, United States), IL-6 using Human IL-6 High sensitive ELISA Kit (eBioscience, Vienna Austria), leptin using SPI-BIO (Montigny Le Bretonneux, France) and adiponectin using ELISA Kit (Sigma-Aldrich Inc., United States); all according to manufacturers’ protocol. Plasma glucose following an OGTT was analyzed at the time of blood sampling. HOMA-IR was calculated using fasting insulin (pmol/L) × fasting glucose (mmol/L) divided by 135 (29, 30).

Mature milk samples were collected as full breast expressions using an electric pump and 250 mL collection bottles (Medela Symphony; Medela; Baar, Switzerland). Samples were collected at the three postpartum visits (V2-V4), and time since last meal of the mother and infant was recorded. From the full breast expression, a 30 mL sample was retained in an amber 50 mL polypropylene tube, and the remaining milk was offered for the mother to take home. Whole milk samples (1.5 mL) were mixed, homogenized and aliquoted into 2 mL amber screw cap tubes immediately after collection and frozen (−80°C) until analysis. Milk samples were analyzed for TNFα, IFNγ, IL-6, IL-8 and the hormones leptin and insulin using MSD U-plex immunoassays (Meso Scale Diagnostics, Rockville, United States). Milk adiponectin was analyzed using sandwich enzyme-linked immunosorbent assay and the human adiponectin duoset (DY1065) from R&D (Biotechne, Minneapolis, MN, United States). Samples were diluted 1:2 for inflammatory markers as well as leptin and insulin, whereas dilution was 1:10 for adiponectin. Assays were performed according to manufacturer protocols. Lower limits of detection were 12 pg./mL (leptin), 11 pmol/L (insulin) and 30 pg./mL (adiponectin), 1.0 pg./mL (TNFα), 3.4 pg./mL (IFNγ), 0.7 pg./mL (IL-6), and 0.3 pg./mL (IL-8). For non-detectable (ND) data, half of the lower cut-off concentration of the specific marker was used for statistical analyses. An internal reference sample was prepared by pooling aliquots of 80 samples and included in duplicates on each plate. The obtained values were used to determine assay variability. The intra assay coefficient of variability (CV) for insulin, leptin and adiponectin was 8.5, 9.5, and 11%, and inter assay CV-values were 18, 20, and 29%, respectively. For TNFα, IFNγ, IL-6, and IL-8, the intra assay CV-values were 28, 10, 18, and 8%, respectively, and inter assay CV-values were 66, 22, 18, 8, and 14%, respectively.

### 2.4. Milk intake

Infant milk intake was estimated at V2-V4 using the 24-h test weighing method and a digital scale (ADE M101000-01; ADE GmbH & Co., Hamburg, Germany) with the accuracy of 5 g for weights <10 kg and 10 g for weights >10 kg. The mothers were instructed to complete the test weighing protocol within the week following each visit by weighing the infants wearing the same clothes before and after each feed for 24 h plus one extra weighing. Total milk intake was defined as intake during the registered period, divided by the number of hours and multiplied by 24. Milk intake per kg bodyweight was estimated by dividing total milk intake with the weight of the infant measured at the visit. Feeds >400 g were regarded as outliers and set to missing, whereas logs with >3 missing feeds were regarded invalid and discarded from analyses. For logs with ≤3 missing feeds, the hot deck imputation method using neighboring weights from the same infant was applied.

### 2.5. Data from obstetric medical files

The following data were obtained from the medical files at the hospital; infant sex (female/male), birthweight (g), date of birth, placenta weight (g), parity (nulliparous/multiparous), mode of delivery (vaginal, elective/acute cesarean section), assisted births (induction, vacuum extraction), use of epidural or oxytocin during birth (yes/no), blood loss at birth (mL), Apgar score at 5 min.

### 2.6. Statistical analysis

Continuous variables are presented as mean ± standard deviation (SD) for normally-distributed data and as median and interquartile range (IQR) for non-normally distributed data. Categorical variables are presented as counts and percentages. Normal distribution of data was checked using histograms and Quantile-Quantile plots prior to analyses. Non-normally distributed data were log-transformed prior to analysis and model estimates were back-transformed to percent change for reporting. Collinearity and equal variance of residuals were checked before reporting model estimates.

Our primary analyses included associations between inflammatory, lipid and metabolic markers (pregnancy markers) and pregnancy and breastfeeding outcomes, respectively, whereas our secondary analyses included assessment of maternal predictors of the pregnancy markers.

Linear regression analysis was applied in both the primary and secondary analyses.

Linear regression analysis was applied in the primary analyses with exposures including the pregnancy markers hs-CRP, TNFα, IFNγ, IL-6, IL-8, HDL, LDL, VLDL, total-cholesterol, triglycerides, leptin, adiponectin, insulin, C-peptide as well as a two-hour OGTT and HOMA-IR, while outcomes included GA at birth, placental weight, birthweight z-score and duration of EBF. Birthweight z-scores were calculated using the INTERGROWTH 21st Study software and thus birthweights were adjusted for gestational age at birth (31). Additionally, linear mixed-effect models (with subject ID as random effect) were used to investigate associations between the pregnancy markers and repeated measures of milk intake per kg bodyweight and HM markers (leptin, insulin, adiponectin, TNFα, IFNγ, IL-6, and IL-8) across lactation. Here, interaction terms between the pregnancy markers and visit as well as the pregnancy markers and infant sex were included to test if the associations between the pregnancy markers and milk intake per kg bodyweight and/or HM markers differed between postpartum visits and infant sex. Milk intake per kg bodyweight was chosen over total milk intake to acknowledge the potential driving effect of infant weight on milk intake (32). Models were made separately for each marker and each outcome of interest, and the following covariates were additionally included in the primary analyses: maternal pre-pregnancy BMI, age and parity. Infant sex was further included when investigating placental weight and HM markers across lactation, while infant age was included when investigating duration of EBF as outcome.

For the secondary analyses, maternal pre-pregnancy BMI, age and parity were included as exposures with each pregnancy marker as outcomes, i.e., separate models for each pregnancy marker.

Covariates was chosen *a priori* based on existing evidence, plausible biological explanations and Directed Acyclic Graphs (DAGs) constructed using dagitty.net (33).

Statistical analyses were conducted using R software (version 4.1.3; R Foundation for Statistical Computing) (34). The *lme4*-package was used to construct linear mixed-effect models. A *p*-values of <0.05 was chosen as the level of significance for additive covariates, whereas *p* < 0.1 were chosen for interactions.

## 3. Results

**Figure 2:**
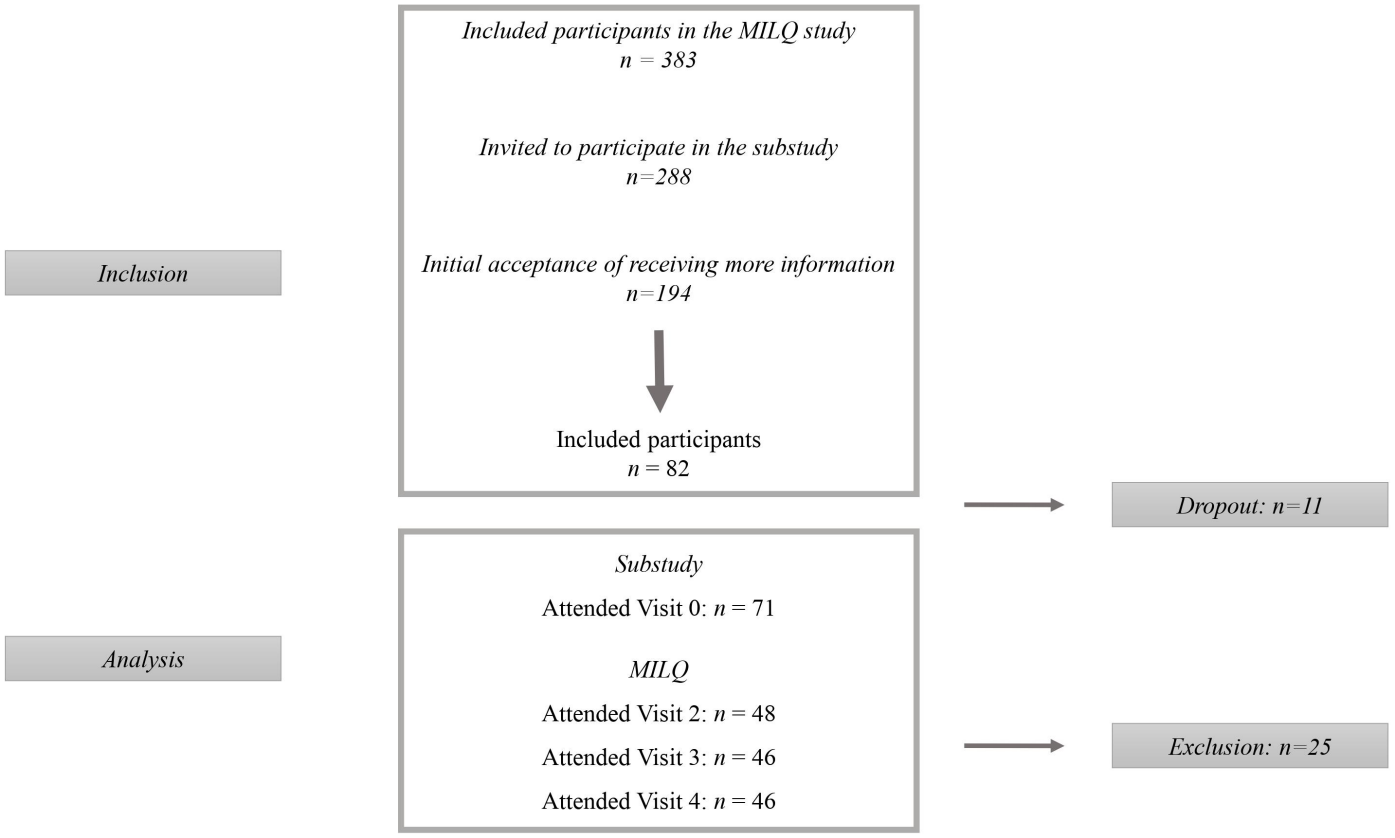
Flow diagram of participants. The present study is a sub-study to the Mothers, Infants, and Location Quality (MILQ) study, where participants were invited to participate after inclusion in the MILQ study.

Out of the 383 mothers enrolled in the MILQ study, 288 were invited to participate in the substudy. Of these, 194 accepted to receive more information and 82 finally accepted participation and were enrolled. Among these, 11 dropped out resulting in 71 completing the study (attended blood sampling during pregnancy, V0) of which 46 also completed the MILQ study (attended physical examination Visits, V2-V4) (Figure 2). The participants who were excluded due to non-EBF at V2 or no BF at V3 did not differ significantly from the participants completing the MILQ study with respect to the inflammatory, lipid or metabolic markers in pregnancy or birth-related characteristics (data not shown).

Mothers had a mean age of 31.3 ± 4.0 years at inclusion and were healthy with a mean pre-pregnancy BMI of 22.9 ± 2.7 kg/m^2^ (Table 1). None of the mothers was clinically diagnosed with GDM (plasma glucose following two-hour OGTT ≥9.0 mmol/L).

**Table 1:** Participant characteristics.

Maternal characteristics		All (*n* = 71)
Age, years		31.3 (4.0)
Pre-pregnancy BMI, kg/m^2^		22.9 (2.7)
Parity		
Nulliparous		55 (77)
Multiparous		16 (23)
Gestational weight gain (kg)^a^		13.6 (4.6)
Maternal educational level^a^		
Short (<3 years)		9 (19)
Medium (3–4 years)		7 (15)
Long (>4 years)		32 (67)
Birth characteristics		
Induction of labor		17 (24)
Use of oxytocin		21 (30)
Use of epidural		10 (14)
Vacuum extraction		7 (10)
Cesarean section		9 (13)
Acute		6 (8)
Elective		3 (4)
Placental weight, grams		646 (148)
Blood loss, mL		508 (320)

Infant characteristics	Males (*n* = 40)	Females (*n* = 31)	All (*n* = 71)
GA at birth, weeks	40.6 [39.6;41.6]	40.6 [38.9;41.1]	40.6 [39.4;41.3]
Premature birth	2 (5)	0 (0)	2 (2.9)
Birthweight, g	3727 (497)	3522 (568)	3638 (535)
Birthweight z-score	0.7 (0.8)	0.6 (1.2)	0.7 (1.0)
Birth length, cm	52.8 (2.5)	51.3 (2.6)*	52.1 (2.6)
Apgar score, 5 min	10 [7;10]	10 [8;10]	10 [7;10]
Duration of EBF, months^a^	4.7 (0.7)	4.5 (1.2)	4.6 (1.0)
Total milk intake, mL			
Visit 2^a^	791 (149)	789 (88)	790 (125)
Visit 3	829 (252)	871 (147)	848 (210)
Visit 4	660 (197)	675 (205)	666 (198)
Milk intake, mL/kg bodyweight			
Visit 2^a^	131 (24)	157 (26)*	142 (28)
Visit 3	107 (32)	128 (24)*	116 (30)
Visit 4	73 (22)	84 (24)	77 (23)

Values are given as mean (SD), median [interquartile range] or counts (%). ^a^Certain data were collected at the postpartum visit, where the sample size was *n* = 48. *Significant differences between sexes. BMI, Body mass index; GA, Gestational age.

Infants were born with a median GA of 40.6 [39.4, 41.3] weeks and a birthweight of 3,638 ± 535 g and with only two born premature (GA 34 and 36). Fifty-six percent were males who were born 1.44 cm [0.21, 2.68] longer than the females, but similar birthweight (*p* = 0.12). Male and female offspring had similar total milk intake (*p* > 0.05), whereas females had a higher milk intake per kg bodyweight compared to males at V2 and V3 (*p* ≤ 0.018) (Table 1).

Mean plasma concentrations of inflammatory, lipid and metabolic markers measured in pregnancy are presented in Table 2 together with medians of human milk concentrations of the respective markers. Human milk concentrations of TNFα, IFNγ, IL-6, IL-8, leptin and adiponectin decreased through lactation (*p* < 0.05), while concentrations of insulin remained constant in models only adjusted for infant sex (Table 2).

**Table 2:** Concentrations of inflammatory, lipid and metabolic markers measured in maternal plasma in gestational week 28–30 and in human milk between 1.0 and 8.49 months postpartum.

	Maternal plasma concentrations		Human milk concentrations^b^		
Pregnancy markers	n	V0 (Gestational week 28–30)	V2 (1–3.49 months)	V3 (3.5–5.99 months)	V4 (6–8.49 months)
Inflammatory					
hsCRP (mg/L)	67	2.7 [0.4;4.0]	–	–	–
TNFa (pg/mL)^a^	69	0.15 [0.15;0.35]	2.1 [1.2;2.3]	2.1 [2.1;2.1]	1.3 [0.4;2.1]^¤^
IFNγ (pg/mL)^a^	70	0.8 [0.8;0.8]	9.9 [4.9;41.2]	3.8 [0.6;5.8]	2.6 [0.6;6.0]^¤^
IL-6 (pg/mL)^a^	69	0.8 [0.5;1.9]	3.6 [2.1;6.4]	1.4 [0.3;2.6]	1.7 [0.5;2.8]^¤^
IL-8 (pg/mL)^a^	70	1.4 [0.9;2.7]	146 [61;221]	133 [99;265]	244 [162;354]^¤^
Lipid					
HDL (mmol/L)	68	2.0 (0.4)	–	–	–
LDL (mmol/L)	68	3.9 (1.0)	–	–	–
VLDL (mmol/L)	68	0.8 [0.7;0.9]	–	–	–
Total cholesterol (mmol/L)	67	6.7 (1.0)	–	–	–
Triglyceride (mmol/L)	68	1.9 (0.5)	–	–	–
Metabolic					
Leptin (ng/mL)^a^	67	25 (14)	0.13 [0.023;0.35]	0.056 [0.013;0.22]	0.052 [0.013;0.15]^¤^
Adiponectin (μg/mL)^a^	68	4.0 (1.1)	0.0027 [0.0022;0.0041]	0.0018 [0.0012;0.0024]	0.0021 [0.0015;0.0030]^¤^
Insulin (pmol/L)^a^	62	63.0 (26.4)	170 [129;210]	155 [120;203]	157 [118;207]
C-peptide (pmol/L)	68	693 (163)	–	–	–
Glucose (mmol/L) (OGTT, *t* = 0)	67	4.4 (0.4)	–	–	–
Glucose (mmol/L) (OGTT, *t* = 120)	67	6.0 (1.1)	–	–	–
HOMA-IR	62	2.1 (0.9)	–	–	–

Concentrations are presented as mean (SD) for normally distributed data and as median [IQR] for non-normally distributed data. ^a^Half the lower detectable concentration has been used for non-detectable data in plasma and/or human milk. ^b^Estimates are based on a reduced sample size (*n* = 41–46) due to fewer completing the MILQ study after birth. ^¤^Significant change across lactation. HDL, High-Density Lipoprotein; HOMA-IR, Homeostasis Model Assessment of Insulin Resistance; hsCRP, high-sensitive C-Reactive Protein; IFNγ, Interferon-γ; IL, Interleukin; IQR, inter-quartile range; LDL, Low-Density Lipoprotein; SD, standard deviation; OGTT, Oral Glucose Tolerance Test; TNFα, Tumor-Necrosis Factor-α; VLDL, Very Low-Density Lipoprotein.

### 3.1. Primary analyses

#### 3.1.1. Inflammatory, lipid, and metabolic markers and birth outcomes

Maternal HDL concentrations were inversely associated with GA at birth and infant birthweight z-score, whereas concentrations of triglycerides and glucose at *t* = 120 were positively associated with birthweight z-score (Table 3). Furthermore, log-hsCRP, log-VLDL and triglycerides were positively associated with placental weight (Table 3). For log-VLDL and log-hsCRP, a 10% increase in VLDL and hs-CRP concentrations resulted in a 2.1 g and 5.0 g increase in placental weight, respectively.

**Table 3:** Associations between inflammatory, lipid and metabolic markers measured in maternal plasma in gestational week 28–30 and birth outcomes.

Pregnancy markers	(a) Gestational age at birth (weeks)			(b) Placental weight (g)			(c) Birthweight z-score		
	*β*	95% CI	*p*-value	*β*	95% CI	*p*-value	*β*	95% CI	*p*-value
Inflammatory									
Log-hsCRP	0.14	[−0.47 to 0.75]	0.65	56.38	[3.72 to 109.03]	**0.04**	−0.09	[−0.45 to 0.26]	0.60
Log-TNFα	0.20	[−0.23 to 0.63]	0.36	−1.02	[−40.05 to 38.01]	0.96	0.20	[−0.06 to 0.46]	0.13
Log-IFNγ	0.05	[−0.26 to 0.37]	0.74	−3.79	[−32.32 to 24.74]	0.79	0.04	[−0.16 to 0.23]	0.69
Log-IL6	−0.18	[−0.61 to 0.25]	0.40	−11.06	[−50.16 to 28.03]	0.57	−0.04	[−0.31 to 0.22]	0.74
Log-IL8	0.19	[−0.21 to 0.59]	0.34	4.95	[−33.52 to 43.41]	0.80	−0.06	[−0.31 to 0.19]	0.64
Lipid									
HDL (mmol/L)	−1.14	[−2.12 to −0.15]	**0.03**	−52.17	[−144.03 to 39.69]	0.26	−0.92	[−1.51 to −0.33]	**<0.01**
LDL (mmol/L)	0.06	[−0.32 to 0.44]	0.76	20.23	[−14.54 to 54.99]	0.25	−0.01	[−0.24 to 0.22]	0.93
Log-VLDL	0.01	[−0.21 to 0.24]	0.90	22.17	[1.90 to 42.43]	**0.03**	0.13	[−0.00 to 0.27]	*0.052*
Total cholesterol (mmol/L)	0.00	[−0.40 to 0.41]	1.00	16.32	[−21.73 to 54.36]	0.39	−0.06	[−0.30 to 0.18]	0.63
Triglyceride (mmol/L)	0.30	[−0.43 to 1.03]	0.42	98.51	[36.94 to 160.08]	**<0.01**	0.60	[0.17 to 1.03]	**<0.01**
Metabolic									
Leptin (ng/mL)	0.00	[−0.03 to 0.04]	0.77	1.18	[−1.82 to 4.18]	0.44	0.01	[−0.01 to 0.03]	0.49
Adiponectin (μg/mL)	0.16	[−0.19 to 0.51]	0.37	−15.87	[−47.50 to 15.76]	0.32	−0.20	[−0.41 to 0.01]	*0.065*
Insulin (pmol/L)	−0.00	[−0.02 to 0.01]	0.73	0.10	[−1.63 to 1.83]	0.91	0.00	[−0.01 to 0.01]	0.73
C-peptide (pmol/L)	0.00	[−0.00 to 0.00]	0.61	0.11	[−0.14 to 0.36]	0.39	0.00	[−0.00 to 0.00]	0.21
Glucose (mmol/L) (OGTT, *t* = 0)	0.46	[−0.54 to 1.45]	0.36	−2.77	[−91.86 to 86.32]	0.95	0.55	[−0.04 to 1.15]	*0.067*
Glucose (mmol/L) (OGTT, *t* = 120)	−0.04	[−0.39 to 0.31]	0.82	−8.98	[−40.17 to 22.21]	0.57	0.22	[0.03 to 0.41]	**0.03**
HOMA-IR	−0.01	[−0.48 to 0.46]	0.97	4.62	[−45.48 to 54.73]	0.85	0.11	[−0.23 to 0.45]	0.51

Linear regression models were adjusted for the following covariates: (a) maternal pre-pregnancy BMI, age and parity; (b) maternal pre-pregnancy BMI, age and parity; (c) maternal pre-pregnancy BMI, age, parity and infant sex. Sample size vary between 62 and 70 depending on available data on plasma concentrations and outcome data. *p*-values in bold indicate significance (*p* < 0.05), and *p*-values in italic indicate borderline significance (*p* < 0.1). HDL, High-Density Lipoprotein; HOMA-IR, Homeostasis Model Assessment of Insulin Resistance; hsCRP, high-sensitive C-Reactive Protein; IFNγ, Interferon-γ; IL, Interleukin; LDL, Low-Density Lipoprotein; OGTT, Oral Glucose Tolerance Test; TNFα, Tumor-Necrosis Factor-α; VLDL, Very Low-Density Lipoprotein.

#### 3.1.2. Inflammatory, lipid, and metabolic markers and breastfeeding outcomes

Higher HDL, insulin, leptin and HOMA-IR were associated with increased duration of EBF by 1.0, 0.02, 0.03, and 0.7 month per unit increase in each marker, respectively (*p*_all_ ≤ 0.048) (Table 4). Furthermore, an interaction was present between visit*total-cholesterol when investigating human milk intake per kg bodyweight as outcome (*p*_tot-chol_ = 0.062) resulting in an increase of 8.5 mL per kg bodyweight for every mmol/L increase in total-cholesterol at V2 only (*p* = 0.046), but not at V3 or V4 (*p* ≥ 0.073) (Table 4). A similar interaction was initially found between visit and insulin in pregnancy (*p* = 0.089) resulting in a lower milk intake per kg bodyweight per pmol/L increase in plasma insulin at V2 (data not shown). However, the interaction as well as association disappeared when adjusting for infant sex.

**Table 4:** Associations between inflammatory, lipid and metabolic markers measured in maternal plasma in gestational week 28–30 and breastfeeding outcomes.

Pregnancy markers	(a) Duration of exclusive breastfeeding (months)			(b) Human milk intake (mL/kg)			(c) Inflammatory and metabolic markers in human milk (conc.)		
	*β*	95% CI	*p*-value	*β*	95% CI	*p*-value	*β*	95% CI	*p*-value
Inflammatory									
Log-hsCRP	0.21	[−0.38 to 0.79]	0.48	−6.81	[−19.70 to 6.07]	0.30	–	–	–
Log-TNFα	−0.06	[−0.51 to 0.39]	0.80	−1.58	[−10.86 to 7.69]	0.74	−0.03	[−0.45 to 0.51]	0.92
Log-IFNγ	0.06	[−0.23 to 0.35]	0.70	1.18	[−4.80 to 7.16]	0.70	−0.20	[−0.51 to 0.11]	0.21
Log-IL6	−0.15	[−0.56 to 0.26]	0.46	0.63	[−8.71 to 9.96]	0.90	−0.11	[−0.50 to 0.28]	0.57
Log-IL8	−0.08	[−0.43 to 0.27]	0.64	−3.06	[−10.77 to 4.65]	0.43	−0.12	[−0.39 to 0.15]	0.39
Lipid									
HDL (mmol/L)	1.03	[0.07 to 2.00]	**0.04**	11.44	[−9.84 to 32.71]	0.29	–	–	–
LDL (mmol/L)	0.06	[−0.26 to 0.38]	0.70	2.79	[−3.97 to 9.55]	0.42	–	–	–
Log-VLDL	0.11	[−0.10 to 0.32]	0.31	1.83	[−2.52 to 6.18]	0.41	–	–	–
Total cholesterol (mmol/L)	0.20	[−0.16 to 0.55]	0.27	8.54	[0.14 to 16.93]	**0.046**	–	–	–
Triglyceride (mmol/L)	0.36	[−0.33 to 1.05]	0.29	4.58	[−10.48 to 19.65]	0.55	–	–	–
Metabolic									
Leptin (ng/mL)	0.03	[0.00 to 0.06]	**0.048**	−0.01	[−0.70 to 0.67]	1.00	0.03	[0.00 to 0.06]	**0.02**
Adiponectin (μg/mL)	−0.08	[−0.37 to 0.22]	0.60	0.86	[−5.21 to 6.93]	0.78	0.05	[−0.17 to 0.28]	0.64
Insulin (pmol/L)	0.02	[0.00 to 0.04]	**0.03**	−0.00	[−0.00 to −0.01]	0.44	−0.00	[−0.01 to 0.00]	0.81
C-peptide (pmol/L)	0.00	[−0.00 to 0.00]	0.12	−0.01	[−0.06 to 0.04]	0.69	–	–	–
Glucose (mmol/L) (OGTT, *t* = 0)	0.50	[−0.43 to 1.42]	0.29	6.75	[−12.03 to 25.52]	0.48	–	–	–
Glucose (mmol/L) (OGTT, *t* = 120)	−0.24	[−0.52 to 0.03]	*0.08*	−0.40	[−6.13 to 5.32]	0.89	–	–	–
HOMA-IR	0.68	[0.17 to 1.20]	**0.01**	1.06	[−9.23 to 11.35]	0.84	–	–	–

Models (a) were linear regression analyses, whereas models (b–c) were linear mixed-effect models with subject ID as random effect. Models were adjusted for the following covariates: (a) maternal age, pre-pregnancy BMI, parity and standardized age at the visit; (b) maternal age, pre-pregnancy BMI and parity and (c) maternal age, pre-pregnancy BMI, parity and infant sex. Sample size vary between 41 and 46 depending on available data on plasma concentrations and outcome data. *p*-values in bold indicate significance (*p* < 0.05), and *p*-values in italic indicate borderline significance (*p* < 0.1). *The estimate is given only for V2 as an interaction was present between total cholesterol and visit. HDL, High-Density Lipoprotein; HOMA-IR, Homeostasis Model Assessment of Insulin Resistance; hsCRP, high-sensitive C-Reactive Protein; IFNγ, Interferon-γ; IL, Interleukin; LDL, Low-Density Lipoprotein; OGTT, Oral Glucose Tolerance Test; TNFα, Tumor-Necrosis Factor-α; VLDL, Very Low-Density Lipoprotein.

### 3.2. Secondary analyses

#### 3.2.1. Predictors of inflammatory, lipid, and metabolic markers

Maternal pre-pregnancy BMI was positively associated with concentrations of log-hsCRP, log-TNFα, C-peptide, insulin, leptin and HOMA-IR in pregnancy (Table 5). For hs-CRP and TNFα, this resulted in an increase of 10% in both TNFα and hs-CRP, respectively, per 1 kg/m*^2^* increase in BMI. Maternal age was negatively associated with leptin and insulin, but positively associated with fasting glucose (Table 5). Parity was not associated with any of the markers.

**Table 5:** Maternal predictors of inflammatory, lipid and metabolic markers measured in gestational week 28–30.

Pregnancy markers	Pre-pregnancy BMI			Maternal age			Parity		
	*β*	95% CI	*p*-value	*β*	95% CI	*p*-value	*β*	95% CI	*p*-value
Inflammatory									
Log-hsCRP	0.10	[0.04 to 0.16]	**<0.01**	−0.00	[−0.05 to 0.04]	0.90	0.36	[−0.04 to 0.76]	*0.076*
Log-TNFα	0.10	[0.01 to 0.18]	**0.03**	0.00	[−0.05 to 0.06]	0.88	0.41	[−0.16 to 0.97]	0.15
Log-IFNγ	0.10	[−0.01 to 0.21]	*0.068*	−0.00	[−0.08 to 0.07]	0.95	−0.07	[−0.80 to 0.66]	0.85
Log-IL6	0.07	[−0.01 to 0.16]	*0.076*	−0.03	[−0.08 to 0.03]	0.38	0.09	[−0.46 to 0.65]	0.73
Log-IL8	0.04	[−0.05 to 0.12]	0.40	−0.04	[−0.10 to 0.02]	0.24	0.21	[−0.36 to 0.79]	0.47
Lipid									
HDL (mmol/L)	−0.02	[−0.05 to 0.01]	0.24	−0.00	[−0.03 to 0.02]	0.82	0.05	[−0.17 to 0.28]	0.63
LDL (mmol/L)	−0.03	[−0.12 to 0.07]	0.57	0.00	[−0.06 to 0.07]	0.93	0.01	[−0.60 to 0.62]	0.98
Log-VLDL	−0.03	[−0.19 to 0.13]	0.72	0.08	[−0.02 to 0.19]	0.13	0.07	[−0.96 to 1.11]	0.89
Total cholesterol (mmol/L)	−0.05	[−0.14 to 0.05]	0.32	−0.01	[−0.07 to 0.06]	0.85	−0.14	[−0.76 to 0.48]	0.66
Triglyceride (mmol/L)	0.03	[−0.02 to 0.07]	0.29	0.01	[−0.02 to 0.04]	0.56	0.00	[−0.31 to 0.32]	0.99
Metabolic									
Leptin (ng/mL)	2.41	[1.31 to 3.51]	**<0.001**	−1.26	[−2.02 to −0.50]	**<0.01**	5.42	[−1.78 to 12.61]	0.14
Adiponectin (μg/mL)	−0.09	[−0.19 to 0.01]	*0.089*	0.00	[−0.06 to 0.07]	0.90	0.07	[−0.58 to 0.72]	0.84
Insulin (pmol/L)	5.04	[2.95 to 7.13]	**<0.001**	−1.74	[−3.20 to −0.28]	**0.02**	−4.65	[−18.72 to 9.43]	0.51
C-peptide (pmol/L)	33.25	[20.38 to 46.12]	**<0.001**	−7.15	[−16.07 to 1.77]	0.11	−14.39	[−98.98 to 70.20]	0.74
Glucose (mmol/L) (OGTT, *t* = 0)	0.02	[−0.02 to 0.05]	0.35	0.03	[0.01 to 0.06]	**0.02**	−0.03	[−0.26 to 0.21]	0.83
Glucose (mmol/L) (OGTT, *t* = 120)	0.07	[−0.04 to 0.17]	0.24	−0.00	[−0.08 to 0.07]	0.92	−0.19	[−0.85 to 0.48]	0.58
HOMA-IR	0.16	[0.09 to 0.24]	**<0.001**	−0.03	[−0.08 to 0.02]	0.21	−0.17	[−0.68 to 0.33]	0.49

Models included all three covariates: maternal pre-pregnancy BMI (kg/m^2^), age (years) and parity (nulliparous vs. multiparous). Sample size vary between 62 and 70 depending on available data on plasma concentrations and outcome data. *p*-values in bold indicate significance (*p* < 0.05), and *p*-values in italic indicate borderline significance (*p* < 0.1). HDL, High-Density Lipoprotein; HOMA-IR, Homeostasis Model Assessment of Insulin Resistance; hsCRP, high-sensitive C-Reactive Protein; IFNγ, Interferon-γ; IL, Interleukin; LDL, Low-Density Lipoprotein; OGTT, Oral Glucose Tolerance Test; TNFα, Tumor-Necrosis Factor-α; VLDL, Very Low-Density Lipoprotein.

## 4. Discussion

In this population of healthy women without obesity, we found significant associations between inflammatory, lipid and metabolic markers measured around the 28th week of pregnancy and pregnancy and breastfeeding outcomes. Several of the metabolic markers were significantly related to the birth outcomes placental weight, gestational age and birthweight z-score, whereas hs-CRP was the only inflammatory marker positively associated with placental weight. Several of the metabolic markers were furthermore positively associated with duration of EBF, while total cholesterol was positively associated with HM intake. Among maternal predictors, maternal pre-pregnancy BMI and age, but not lipid markers, were associated with certain inflammatory and metabolic markers.

### 4.1. Inflammatory, lipid, and metabolic markers and birth outcomes

Higher plasma HDL in pregnancy was associated with lower GA at birth as well as birthweight z-score, whereas higher triglyceride levels were associated with higher placental weight and birthweight z-score. Similarly, Okala and colleagues found lower plasma triglyceride levels in gestational week 30 among mothers who gave birth to small-for-gestation (SGA) infants in rural Gambia (35). However, the authors found lower HDL in early and late pregnancy associated with a greater risk of giving birth to infants with low birthweight (LBW), which is contrary to our findings. Similarly, higher HDL has been associated with longer duration of pregnancy among mothers in Ghana (36), which is also contrary to our findings. Furthermore, mothers with infants born SGA and LBW in The Gambia also had lower BMI and lower gestational weight gain (GWG). A study by Ouyang et al. reported increased birthweight z-score among mothers with pre-pregnancy BMI ≥30 kg/m^2^ compared to 18.5–24.9 kg/m^2^ and among mothers with excessive compared to adequate GWG according to the Institute of Medicine guidelines (37). The authors found that associations attenuated when they adjusted for placental weight and suggest that the placenta might have a mediating effect on the association. Our findings could similarly indicate a mediated effect of placental weight in the positive associations between triglycerides and birthweight z-score. These and our results may reflect a dietary pattern of high-fat and/or high-carbohydrate intakes, which could affect both lipid profile, GWG, and thereby placental weight followed by increased intrauterine growth. Lastly, the conflicting results found in low-and middle-income countries and in the present study could reflect environmental and genetic differences. However, these suggestions are speculative and were not investigated in the present study.

In line with other studies, we found a positive association between two-hour glucose concentrations following an OGTT and birthweight z-score. This association is especially well-documented in studies of mothers with GDM and obesity (38–40). Yuan et al. found maternal factors such as pre-pregnancy BMI, GWG, glucose values at OGTT, HDL and LDL together with other metabolites predicted macrosomia infants in mothers with GDM (41). Although mothers in the present study did not have obesity or GDM, our findings could indicate similar mechanisms occurring across a wider range of maternal weight statuses in our population.

### 4.2. Inflammatory, lipid, and metabolic markers and breastfeeding outcomes

We found a positive association between total-cholesterol and milk intake per kg bodyweight at V2 and V3, but not at V4. Initially, an inverse associations was found between plasma insulin and milk intake per kg bodyweight at V2 only, but the association disappeared when adjusting for infant sex. Our results may support the findings from a case–control study including 42 mothers, where markers of metabolic health were reported to be worse in mothers with very low milk output (<300 mL/day) compared to nested controls (milk output ≥300 mL/day) and an external control group consisting of exclusively breastfeeding mothers (mean milk output 758 g/day) (42). HOMA-IR, BMI, fasting plasma concentrations of glucose, insulin and C-peptide were higher, whereas concentrations of triglycerides, HDL and prolactin were lower in mothers with extreme low milk output compared to the other groups. Although the sample size in the case–control study is low, these and our results indicate that poorer metabolic health and hormonal imbalance during pregnancy could affect milk production possibly through delayed *lactogenesis II* (26, 43–45). Nommsen-Rivers et al. further showed in a randomized controlled trial, that milk production increased by 60% when intervening with metformin compared to an increase of 20% in the placebo group, although the results were not significant (46). Improvement of milk production correlated strongest with earlier time after delivery and lower baseline milk production, although these results were also non-significant. This may indicate that improvement of insulin sensitivity could increase milk production, and that a stronger effect is seen in mothers with lower milk output early after delivery. As mentioned, associations in the present study attenuated when adjusting for infant sex. As males had a significantly lower intake per kg bodyweight, due to a greater weight than females, this difference between the sexes might drive the association between pregnancy markers and infant milk intake. As the sample size is rather small, it is possible that a small group of males with particularly low milk intake per kg bodyweight had mothers with high insulin in pregnancy, which could drive the association. Generally, estimates of milk intake per kg bodyweight in the present study were comparable to estimates recently published in a systematic review and meta-analysis (32), and are therefore likely to be valid. Lastly, lipid metabolism of the mammary glands during various conditions were not investigated and may be an important explanation for altered HM synthesis.

Our results further showed that plasma HDL, insulin, leptin and HOMA-IR during pregnancy were positively associated with duration of EBF. As higher insulin was initially associated with lower milk intake, and lower milk production may shorten the duration of EBF (47), the results are contrary to the expected. However, the participants were well-educated and motivated to breastfeed, which enhances the chances of successfully establishing and continuing breastfeeding. In addition, the mothers were offered breastfeeding counseling throughout the project period to support breastfeeding recommendations. It is plausible that mothers with breastfeeding complications, possibly due to overweight or altered metabolic profiles, might have used the counselors more and thereby overcame any complications resulting in longer duration of EBF.

Finally, plasma leptin in pregnancy was positively associated with HM concentrations postpartum. Similar findings for adiponectin were reported by Ley et al., who found positive associations between serum adiponectin measured in pregnancy and HM adiponectin at both 2 and 95 days postpartum (24). Other studies have shown positive associations between both pre-pregnancy BMI and plasma leptin, respectively, and HM leptin (48, 49), hence our results confirm previous findings.

### 4.3. Predictors of inflammatory, lipid, and metabolic markers

Our findings indicate that higher pre-pregnancy BMI and younger maternal age were the main contributors to elevated levels of inflammatory and metabolic markers in pregnancy. In addition, the positive association between hs-CRP and placental weight might be explained by increased pre-pregnancy BMI, which has been shown previously (50). However, concentrations of inflammatory and metabolic markers were similar to those in healthy pregnancies (51–53).

The lack of associations between pre-pregnancy BMI and lipid markers may seem surprising as free fatty acids (FFAs) secreted from the excessive adipose tissue are transported to the liver resulting in increased synthesis of triglycerides and VLDL particles, partly in favor of HDL (54, 55). However, as we do not have information on dietary intake or physical activity level at the time of blood sampling, which may have varied substantially depending on the condition of the pregnancy, these factors could have affected plasma lipid concentrations (56, 57). In non-pregnant individuals, higher intakes of, e.g., saturated fatty acids have been associated with higher LDL-cholesterol through increased hepatic LDL secretion and reduced LDL clearance (58), whereas exercise has been shown to increase HDL-cholesterol (59). Additionally, fish oil supplementation was allowed during the project period, which may have reduced triglyceride levels and increased HDL cholesterol (60, 61). Combined with our findings regarding birth and breastfeeding outcomes, it is likely that women who took fish oil supplements were the same women with certain dietary intakes and physical active lifestyles, which contributed to enhanced lipid profiles as well as to a lower placental weight, birth weight z-score, and longer duration of EBF. However, lipid concentrations increase during healthy pregnancies as a results of increased estrogen levels (62–64), and our results were considered within the normal range for pregnant women.

Furthermore, increased FFA secretion from excessive adipose tissue impair insulin sensitivity resulting in reduced glucose uptake in the muscles and a compensatory increase in pancreatic insulin secretion to maintain normoglycemia (16, 65). This mechanism might be reflected in our results showing higher pre-pregnancy BMI was associated with increased C-peptide, insulin and HOMA-IR, but not plasma glucose concentrations. This indicates that women with moderate overweight may be slightly insulin resistant, measured by HOMA-IR, but additionally compensate by having increased insulin production, measured by insulin and C-peptide. C-peptide is often used as a marker of pancreatic insulin secretion, as it is secreted into the plasma in equimolar amounts as insulin, while also having a longer half-life than insulin making it a more stable marker than plasma insulin (66). However, C-peptide concentrations in the present study are within the normal range of healthy adults, which indicate normal pancreatic insulin secretion as expected. Insulin resistance has further been reported in healthy pregnancies with a reduction in insulin sensitivity up to 27% in the third trimester (67), and it was not determined if the reduction in sensitivity in the present study was within normal range.

Positive associations were found between pre-pregnancy BMI and plasma leptin, which was expected as the adipokines leptin is secreted from adipose tissue (68). Furthermore, leptin secretion is stimulated by increased insulin levels (69), which could be a contributing mechanism in the present study for women with slight insulin resistance.

Finally, maternal age was inversely associated with leptin and insulin, also when adjusting for parity, but positively associated with fasting glucose although effect estimates were small. The latter was expected, as hyperglycemia and insulin resistance increase with age (70).

### 4.4. Strengths and limitations

The main strength of our study is the data collection covering both pregnancy as well as breastfeeding, and interesting results were found even in healthy mothers with normal-weight and slight overweight. However, factors related to pregnancy may influence the infant both in the short-term, e.g., birthweight and gestational age, but also in the longer term, e.g., through breastfeeding. The challenge lies in disentangling the influence of pregnancy on short-term outcomes from the influence of pregnancy on the long-term outcomes. It is likely that a series of mechanisms affect each other, whereby the outcome of interest is affected cumulatively. As only a few of these mechanisms are confirmed in the literature, caution must be taken when statistically analyzing data, especially to avoid retrieving biased estimates (71, 72). The use of birthweight z-score compared to using birthweight might seem less clinically relevant as most of the infants were born at term. However, as GA is likely to affect birthweight, despite a term birth, GA was considered relevant to adjust for. Adjusting separately for GA was considered inappropriate as GA might be mediating the influence on birthweight, and thus, birthweight z-score was chosen in analyses.

The study holds certain limitations of which the sample size is of most concern as 71 participants were included and only 46 completed the postpartum study. The study might be underpowered to confirm the results, especially regarding outcomes measured in the postpartum period. Furthermore, the effect estimates are relatively small with wide confidence intervals. These aspects reduce the external validity of the findings and the study should be replicated in a larger population in order to increase generalizability of the results. In addition, *n* = 3 had ceased EBF before the age of 3 months, while *n* = 2 were born prematurely (GA < 37 + 0). It is possible that certain associations were driven by a few participants in this small cohort. It could further be relevant to investigate the associations within the groups of normal-versus overweight to support the findings. However, this would require a larger sample size with evenly distributed groups. Furthermore, plasma concentrations of the inflammatory markers TNFα and IFNγ were below the detection limit for 72 and 76% of the samples, respectively. Half of the lower cut-off concentration was therefore used in analyses, which may have resulted in uncertain estimates. In that regards, assays used for HM analyses have not been validated in the HM matrix, neither in the present study nor in existing literature, and thus estimates of HM concentrations might have been affected, whereas associations are less likely to be affected. The high number of analyses additionally introduces a risk of chance findings. Applying correction for multiple testing, e.g., Bonferroni correction, could reduce this risk, however, this was omitted for the explorative purpose of the study. The strength of using several markers for, e.g., lipid profile is the possibility of finding consistent results across several markers. Each marker adds valuable information individually and, when combined, strengthens the findings and thereby the understanding of the underlying mechanisms.

Lastly, it is worth reiterating that the population was healthy pregnant women without obesity. Although certain associations were significant, the effect estimates were small, thus the clinical relevance can be questioned. However, the findings might be of relevance in other populations where associations might be stronger and/or estimates larger.

## 5. Conclusion

We showed that maternal metabolism during pregnancy was associated with several important birth-related and breastfeeding outcomes in this relatively small cohort of healthy Danish women. Mainly lipid markers were associated with birth outcomes such as birthweight z-score, whereas higher metabolic markers were associated with longer duration of exclusive breastfeeding.

Finally, pre-pregnancy BMI was the main predictor of metabolic markers involved in glucose homeostasis and insulin resistance, which is in accordance with current literature.

Despite the fact that the estimates are marginally significant, the findings provide information that can help to understand mechanisms behind early programming and thereby optimize short-and long-term health of infants. However, further studies are encouraged to confirm the findings and explore the pathways by which the associations occur.

## Data availability statement

The datasets presented in this article are not readily available due to them containing information that could compromise research participant privacy/consent. Further inquiries can be directed to the corresponding author. Requests to access the datasets should be directed to SC, sch@nexs.ku.dk.

## Ethics statement

The studies involving humans were approved by the Ethics Committee of Capital Region of Denmark. The studies were conducted in accordance with the local legislation and institutional requirements. Written informed consent for participation in this study was provided by the participants’ legal guardians/next of kin.

## Author contributions

LA, KM, SC, CM, AR, TG, and KR participated in designing and conducting the study. HF analyzed HM samples. SC and JL analyzed data statistically and wrote the manuscript. SC had primary responsibility for the final content. All authors have read and approved the final manuscript.

## Funding

This work was supported by the Bill & Melinda Gates Foundation (grant nos. OPP1148405 and INV-002300), intramural USDA-Agricultural Research Service project 5306–51000-004-00D, the University of Copenhagen (salary) and *Læge Sofus Carl Emil Friis og Hustru Olga Doris Friis Fond* (collection and analysis of blood samples and OGTTs in pregnancy). USDA was an equal opportunity employer and provider.

## Conflict of interest

The authors declare that the research was conducted in the absence of any commercial or financial relationships that could be construed as a potential conflict of interest.

## Publisher’s note

All claims expressed in this article are solely those of the authors and do not necessarily represent those of their affiliated organizations, or those of the publisher, the editors and the reviewers. Any product that may be evaluated in this article, or claim that may be made by its manufacturer, is not guaranteed or endorsed by the publisher.

## Figures and Tables

**Table:** 

BMI	Body mass index
CI	Confidence interval
CV	Coefficient of variance
DAGs	Directed acyclic graphs
EBF	Exclusive breastfeeding/Exclusively breastfed
GA	Gestational age
GDM	Gestational diabetes mellitus
HDL	High-density lipoprotein
HOMA-IR	Homeostasis model assessment of insulin resistance
HM	Human milk
Hs-CRP	High-sensitivity C-reactive protein
IFNγ	Interferon gamma-γ
IL	Interleukin
IQR	Inter quartile range
LDL	Low-density lipoprotein
MILQ	“Mothers, Infants and Lactation Quality” study
ND	Non-detectable
OGTT	Oral glucose tolerance test
PBF	Partial breastfeeding
SD	Standard deviation
TNFα	Tumor-necrosis factor-α
VLDL	Very low-density lipoprotein
V2, V3, V4	Visit 2, Visit 3, Visit 4
WHO	World Health Organization
WLZ	Weight-for-length Z-scores
